# Whole slide images reflect DNA methylation patterns of human tumors

**DOI:** 10.1038/s41525-020-0120-9

**Published:** 2020-03-10

**Authors:** Hong Zheng, Alexandre Momeni, Pierre-Louis Cedoz, Hannes Vogel, Olivier Gevaert

**Affiliations:** 1 Stanford Center for Biomedical Informatics Research (BMIR), Department of Medicine, Stanford University, Stanford, USA; 2 Department of Pathology, Stanford University, Stanford, USA; 3 Department of Biomedical Data Science, Stanford University, Stanford, USA

**Keywords:** Cancer, Computational biology and bioinformatics, Cancer imaging, CNS cancer

## Abstract

DNA methylation is an important epigenetic mechanism regulating gene expression and its role in carcinogenesis has been extensively studied. High-throughput DNA methylation assays have been used broadly in cancer research. Histopathology images are commonly obtained in cancer treatment, given that tissue sampling remains the clinical gold-standard for diagnosis. In this work, we investigate the interaction between cancer histopathology images and DNA methylation profiles to provide a better understanding of tumor pathobiology at the epigenetic level. We demonstrate that classical machine learning algorithms can associate the DNA methylation profiles of cancer samples with morphometric features extracted from whole slide images. Furthermore, grouping the genes into methylation clusters greatly improves the performance of the models. The well-predicted genes are enriched in key pathways in carcinogenesis including hypoxia in glioma and angiogenesis in renal cell carcinoma. Our results provide new insights into the link between histopathological and molecular data.

## Introduction

DNA methylation is an important epigenetic mechanism regulating various biological processes. DNA hyper-methylation and hypo-methylation are important mechanisms that deregulate gene expression in a wide range of cancers. Aberrant DNA methylation is one of the most common and well-studied molecular alterations in cancer and DNA methylation changes have emerged as important biomarkers and epigenetic drivers of cancer.^1–5^ High-throughput DNA methylation assays are being used more frequently in cancer research, generating vast amounts of genome-wide DNA methylation measurements. For example, the Cancer Genome Atlas (TCGA) project generated a rich source of epigenomic data for cancers of various organs,^6^ including the profiling of DNA methylation using microarray technology in over 10,000 samples. Several approaches have been developed to profile DNA methylation pattern in cancers and identify differentially methylated genes from DNA methylation profiling assays. For example, we have developed MethylMix, a beta mixture model-based method that identifies DNA methylation driver genes in cancer.^7–10^ MethylMix integrates DNA methylation and gene expression data from normal and disease samples and identifies differentially methylated genes that are also predicative of gene expression levels. The main output of MethylMix are the “Differential Methylation” values or DM-values, defined as the difference between an abnormal methylation state (e.g., hyper-methylated or hypo-methylated) and the normal methylation state. MethylMix has been used to identify methylation driver genes and reveal cancer subtypes across heterogeneous samples. For example, in head and neck cancer, we discovered five distinct DNA methylation subtypes identified by DM values differing from previously reported gene expression subtypes. These DNA methylation subtypes better segregate with etiological subgroups defined by HPV status and smoking in head and neck cancer.^11^ A similar hypo-methylated subtype defined by NSD1 inactivation was also identified across squamous cell carcinoma.^12^ Furthermore, proteomic data can also be leveraged in the MethylMix analytical framework to further narrows down the candidate methylation driver genes in cancer.^13^

DNA methylation holds great promise as biomarkers in cancer, however, generating DNA methylation data of cancer patients has yet to become common clinical practice, since the performance of DNA methylation biomarkers varies across diseases and remains to be evaluated and improved, which is an active area of research. Besides, the turnaround time for results may take several weeks, delaying important therapeutic decisions. In contrast, whenever a patient is suspected to have cancer, whole slide images are often available from tissue sections, which provide a wealth of information about the tissue architecture and cell composition. Additionally, the adoption of digital pathology has led to great advances in the storage of whole slide images and subsequently has spurred the field of automated computational analysis of these images. For example, the C-Path (Computational Pathologist) system was developed to quantify a rich feature set from whole slide images of breast cancer epithelium and stroma. These features were then used to construct a prognostic model which predicted overall survival and identified features of prognostic relevance. An new finding from this study was that the features that were the best predictors of patient survival were not from the cancer itself but were from the adjacent stromal tissue.^14^ Another study combined histology images of tissue biopsies and genomic biomarkers to predict the survival of glioma patients using convolutional neural networks.^15^ Furthermore, to address the large technical and biological variations that are always present in a large cohort, several algorithms such as spatial pyramid matching (SPM) were proposed to constructed morphometric context from nuclear morphometric statistics of various locations and scales.^16^

Although molecular information such as DNA methylation and histology information from whole slide images can potentially be used in clinical practice, more research and evaluation needs to be performed to address any clinical utility and methodological difficulties. In addition, more insight into the relationship between molecular and imaging features are necessary to integrate them for clinical decision support. Therefore, we aim to understand whether and how DNA methylation patterns are reflected in whole slide images. We apply classical machine learning techniques to predict DNA methylation patterns of cancer patients using morphometric features extracted from whole slide images. We show results in two different cancer sites, glioma and renal cell carcinoma (RCC).

Gliomas are a heterogeneous group of tumors of the central nervous system, which are thought to derive from neuroglial stem or progenitor cells and are responsible for the majority of deaths from primary brain tumors. They are classified histologically into astrocytic, oligodendroglial, or ependymal tumors. Based on different degrees of malignancy, gliomas are assigned World Health Organization (WHO) grades, including grades II and III lower-grade gliomas (LGG), and glioblastomas (GBM), the highest-grade gliomas. GBM is distinguished histopathologically from LGG by the presence of necrosis or microvascular proliferation.^17–19^ Gliomas demonstrate high variability in phenotype, genotype, epigenotype, clinical course, therapeutic response, and outcome. This heterogeneity is in part attributed to the diverse genetic and epigenetic alterations that occur early in tumorigenesis. Enormous progress in genomic, transcriptomic, and epigenetic profiling has resulted in new concepts of classifying and treating gliomas.^18,19^ The WHO has recently placed new emphasis on the integration of both histopathological and molecular data for the classification of gliomas to improve disease understanding and to guide personalized therapies.^20^

Next, RCC are derived from renal tubular epithelial cells and encompasses a large heterogeneous group of cancers, representing more than ten molecular and histopathological subtypes and accounting for more than 90% of cancers detected in the kidney. RCC are traditionally divided on the basis of morphological features into clear cell, papillary, chromophobe, collecting duct, and unclassified renal cell carcinoma. The clear cell subtype of RCC is the most common subtype.^21–23^

**Fig. 1:**
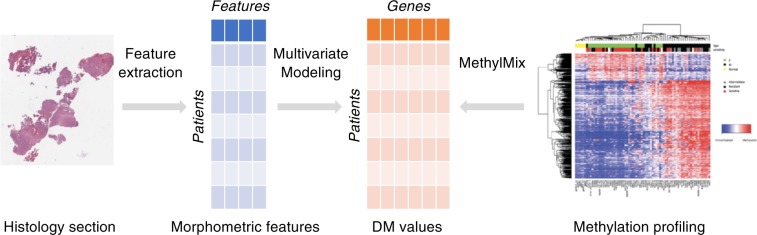
Representation of the machine learning pipeline to predict DNA methylation states from whole slide images. The predictors are the morphometric features extracted from the whole slide images. The labels are the differential methylation (DM) values computed from DNA methylation data using MethylMix.

Here, we present the results of DNA methylation prediction in glioma and RCC samples based on morphometric features extracted from whole slide images (Fig. 1). The performance of the model is greatly improved using gene clustering. Top biological pathways are identified through gene set enrichment analysis. Our results underline the potential of using whole slide images to predict DNA methylation states, providing new insights into the link between histopathological features and molecular data.

## Results

### Morphometric features predict gene methylation states of glioma patients

**Fig. 2:**
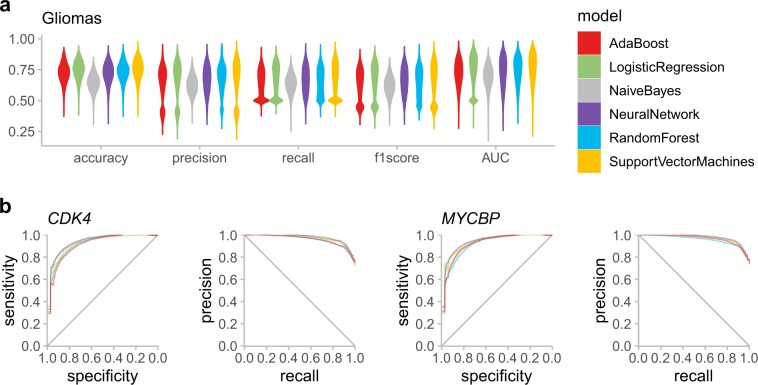
Gene-level methylation prediction for the glioma samples. **a** The distribution of five evaluation metrics for each machine learning model is shown by the violin plot. **b** The receiver operating characteristics (ROC) curve and precision-recall (PR) curve for two genes whose methylation states are well-predicted by morphometric features in the glioma samples. AUC area under the curve.

First, we used MethylMix on a glioma cohort of 342 samples to identify differential methylated genes serving as the most important epigenomic biomarkers for glioma. This resulted in 927 genes reported by Methylmix as differentially methylated and predictive of matched gene expression data. Out of the six models we tested, all classifiers have an average area under the curve (AUC) score above 0.7, except for the naïve Bayes classifier, which was excluded from subsequent analysis (Fig. 2a). The average AUC and *F*1 score across all genes in the remaining five models are 0.74 and 0.64, respectively. Out of the 927 genes, eight of them (*CDK4, COL5A1, CPA4, CSTB, MMP7, MYCBP, PPIC, TMEM59*) have an average AUC and *F*1 score higher than 0.8 under all the machine learning models (Fig. 2b).

Cyclin Dependent Kinase 4 (CDK4), a member of the Ser/Thr protein kinase family, is a key player in cell cycle progression (G1 to S phase) and is implicated in the tumorigenesis of a variety of cancers. It is also the focus of cancer therapeutic research and development.^24,25^ It is among the most frequently altered genes in gliomas.^26^ MYC binding protein (MYCBP) encodes a protein that binds to the oncogenic protein C-MYC. It is normally found in the cytoplasm, but it translocates to the nucleus during S phase of the cell cycle. Overexpression of this gene was shown to promote invasion and migration in gastric cancer.^27^ Both genes are involved in the cell cycle progression and the methylation states of them are well-predicted by morphometric features in the glioma samples. Both *CDK4* and *MYCBP* have an AUC score over 0.94 and *F*1 score over 0.84 under logistic regression, support vector machines, and neural network models.

Transmembrane Protein 59 (TMEM59), also known as dendritic cell factor 1 (DCF1), was involved in the differentiation of neural stem cells. Over-expression of this protein has been found to promote apoptosis in a glioma cell line.^28^ MMP7 (matrix metallopeptidase 7) belongs to the matrix metalloproteinase family that are involved in the expansion and invasion of gliomas via extracellular matrix degradation.^29,30^ The methylation states of them are also well-predicted by morphometric features in the glioma samples. *TMEM59* has an AUC over 0.91 and *F*1 score over 0.82 under all models. *MMP7* has an AUC of 0.92 and *F*1 score of 0.85 under the support vector machines model.

### Gene clustering using DM-values improves model performance

**Fig. 3:**
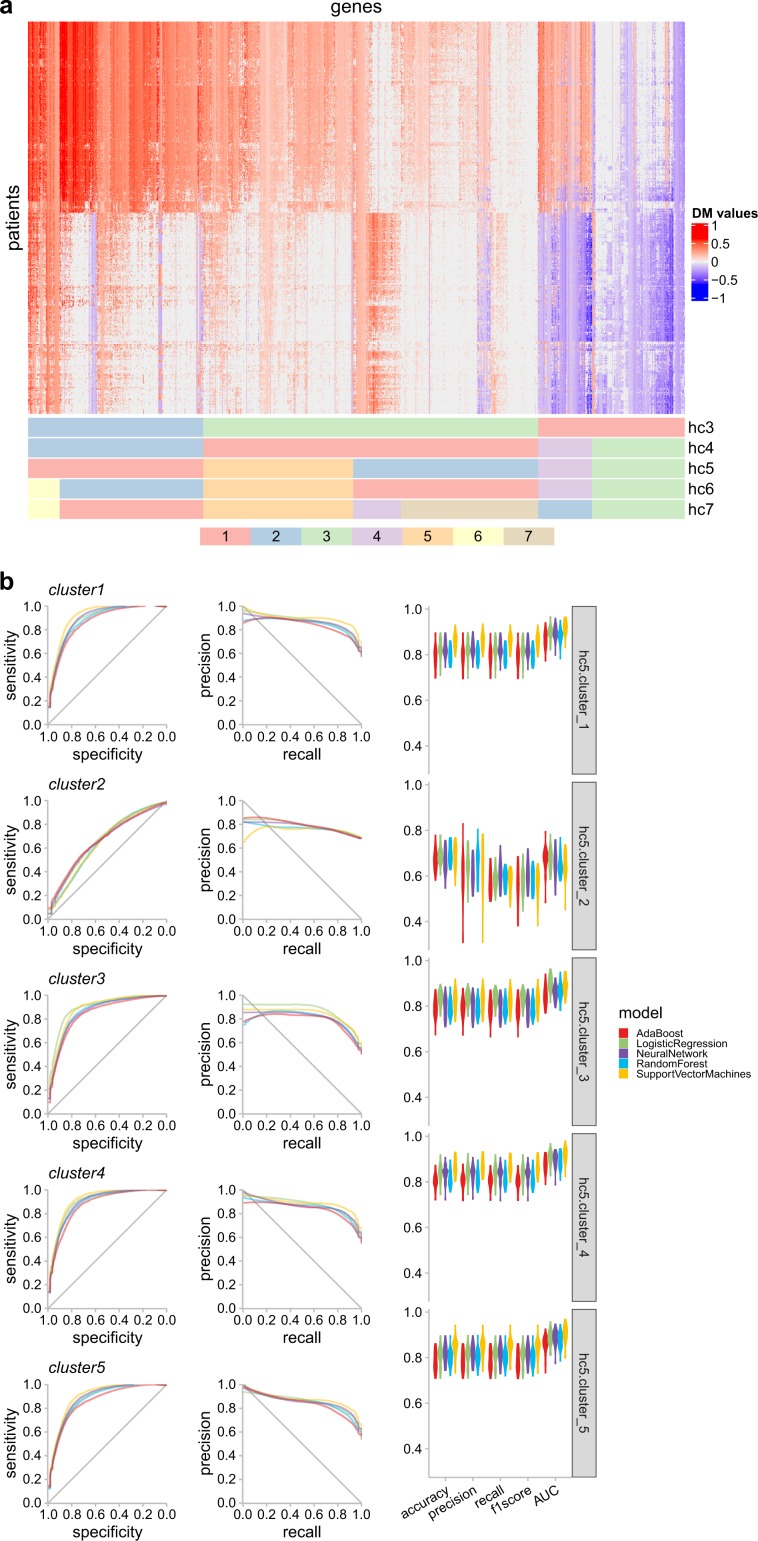
Cluster-level methylation prediction for the glioma samples. **a** Heat map of the hierarchical clustering visualization of the glioma samples. The column corresponds to the genes and the row corresponds to the patients. Cluster assignments of the genes are shown in the bottom annotation panel, from three clusters (hc3) to seven clusters (hc7). **b** The receiver operating characteristics (ROC) curve and precision-recall (PR) curve of the prediction tasks of five clusters (hc5) and the distribution of the corresponding evaluation metrics by violin plots.

Next, we clustered the DM-values of all MethylMix genes into methylation clusters, so that genes with similar DNA methylation status are grouped together. We chose five clusters from the hierarchical clustering results based on visual inspection of the clustering results (Fig. 3a) and the Silhouette score analysis (Supplementary Fig. 1). The cluster-level methylation states were summarized from the DM-values of the genes in the cluster and discretized by an optimal threshold determined using a Gaussian mixture model.

Next, we predicted the cluster-level DNA methylation states with morphometric features. Grouping genes into clusters was shown to considerably improve the performance of the models, compared to single gene-level prediction (Fig. 3b). Except for cluster 2, all other clusters have an AUC and *F*1 score larger than 0.8.

### Fewer genes and clusters can be predicted by morphometric features for RCC

**Fig. 4:**
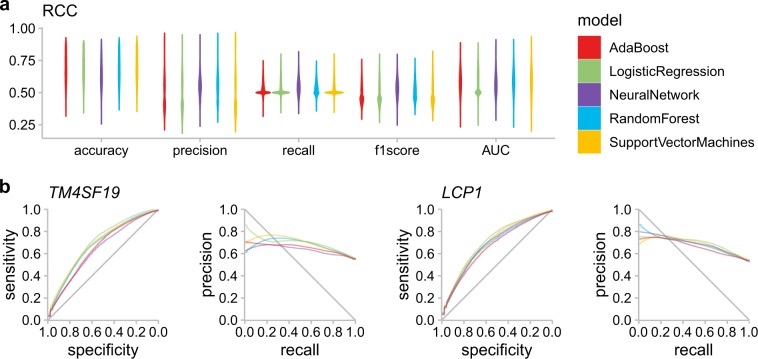
Gene-level prediction in the RCC samples. **a** The distribution of five evaluation metrics for each machine learning model is shown by the violin plot. **b** The receiver operating characteristics (ROC) curve and precision-recall (PR) curve for two genes whose methylation states are well-predicted by morphometric features in the RCC samples. AUC area under the curve.

Next, we tested whether DNA methylation patterns can be predict in RCC. Similar as for the glioma cohort, we first used MethylMix on 326 RCC samples where both molecular data and morphometric features are available. After applying MethylMix, 366 genes were identified as differentially methylated in the RCC cohort. The overall AUC and *F*1 score are lower compared to the glioma cohort (Fig. 4a). The average AUC score across all genes for the five models is 0.58.

Out of the 366 genes, five of them (*DAK, ITPRIP, LCP1, TM4SF19, TMEM200A*) have an average AUC and *F*1 score higher than 0.6 under all the models (Fig. 4b). LCP1 (Lymphocyte Cytosolic Protein 1) is one of the two ubiquitous plastin isoforms identifed in human. LCP1, the L isoform has been found in many types of malignant human cells of non-hemopoietic origin. It is identified as one of the three biomarkers that identify early stage kidney cancer.^31^ *LCP1* has an AUC over 0.7 and *F*1 score over 0.65 under logistic regression and support vector machines models.

**Fig. 5:**
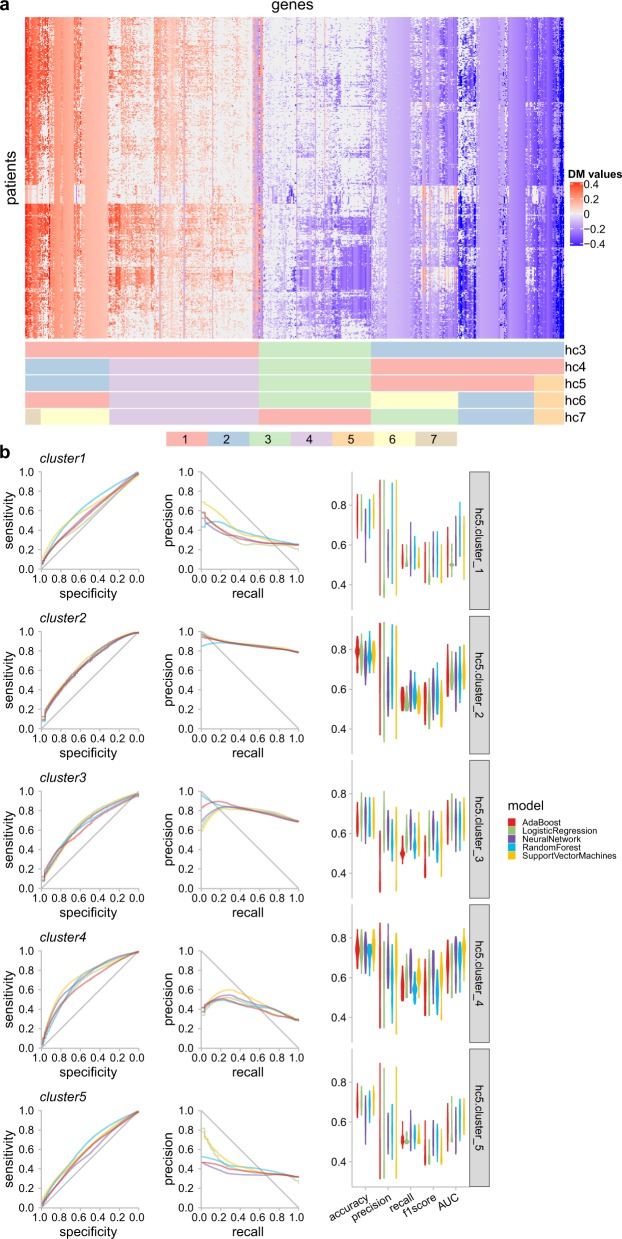
Cluster-level prediction in the RCC samples. **a** Heat map of the hierarchical clustering visualization of the RCC samples. The column corresponds to the genes and the row corresponds to the patients. Cluster assignments of the genes are shown in the bottom annotation panel, from three clusters (hc3) to seven clusters (hc7). **b** The receiver operating characteristics (ROC) curve and precision-recall (PR) curve of the prediction tasks of five clusters (hc5) and the distribution of the corresponding evaluation metrics by violin plots.

Similar as for the glioma cohort, gene clustering of the DM values improves the model performance (Fig. 5). The average AUC and *F*1 score for three clusters (cluster 2, 3, and 4) in hierarchical clustering (number of clusters were set at 5) exceed 0.66 and 0.54, respectively.

### Gene set enrichment analysis

For the glioma set, we divided the genes into two groups, 321 genes with AUC and F1 scores above 0.7 (GoodPredSet) in at least 4 models, and 45 genes with AUC and F1 score less than 0.6 in all the models tested (BadPredSet). We performed gene set enrichment analysis using the Hallmark gene sets from the Molecular Signatures Database.^32^ The GoodPredSet genes are enriched in apoptosis and hypoxia pathway, while none of these pathways is enriched in the BadPredSet genes (Table 1). Hypoxia is a condition in which an organism or a cell is deprived of adequate oxygenation. It constitutes a major concern for glioma patients, since it promotes cancer cell invasion into the healthy brain tissue in order to evade this adverse microenvironment.^33^ Exposure to hypoxia was shown to change in cell morphology and enhanced invasive capacity of glioblastoma cells.^34^

**Table 1:** Gene set enrichment analysis.

Gene set	Gene set size	*p* value	Adjusted *p* value	Overlapping genes	Cancer
Apoptosis	161	1.17e–05	1.17e–04	*BIRC3; CASP8; TNFSF10; FAS; CCND1; BTG3; LGALS3; LMNA; CD2; MMP2; WEE1; ERBB2*	Glioma
Interferon gamma response	200	2.64e–08	1.32e–06	*MX1; MX2; TNFSF10; IFI44; BST2; IFIH1; LY6E; C1S; ITGB7; SOCS1; CIITA; CASP8; MT2A; UPP1; LATS2; FAS; APOL6*	Glioma
Hypoxia	200	2.22e–05	1.39e–04	*GBE1; LDHA; LOX; F3; PTRF; MT1E; MT2A; ANXA2; PGAM2; PAM; PLAC8; FBP1; COL5A1*	Glioma
Inflammatory response	200	2.22e–05	1.39e–04	*CCR7; TNFSF10; LYN; F3; BST2; CD48; RGS16; SLAMF1; LY6E; PDPN; MET; MSR1; EMP3*	Glioma
Xenobiotic metabolism	200	2.22e–05	1.39e–04	*CYP27A1; AOX1; CA2; FBP1; VNN1; CROT; TMEM176B; MT2A; TNFRSF1A; UPP1; CBR1; EPHA2; FAS*	Glioma
Epithelial mesenchymal transition	200	1.58e–07	2.63e–06	*COL5A2; COL5A1; LOX; MMP2; FAP; LOXL1; FBLN5; IL32; EMP3; MGP; PCOLCE; EFEMP2; FMOD; SFRP4; FAS; ADAM12*	Glioma
KRAS signaling up	200	1.58e–07	2.63e–06	*BIRC3; SLPI; TMEM158; MMP9; ITGBL1; TRIB2; TMEM176B; PDCD1LG2; CA2; RGS16; FBXO4; CROT; F13A1; EPB41L3; ENG; GPNMB*	Glioma
Coagulation	138	7.96e–05	4.42e–04	*C1S; MMP2; MMP9; F2RL2; PRSS23; F3; CSRP1; SERPINA1; APOC2; MMP7*	Glioma
UV response DN	144	3.72e–06	4.65e–05	*ABCC1; MET; ANXA2; RUNX1; LTBP1; COL5A2; F3; ERBB2; EFEMP1; TGFBR2; FBLN5; MT1E*	Glioma
IL2 STAT5 signaling	200	1.52e–03	6.89e–03	*SOCS1; CD83; ST3GAL4; RGS16; BATF; CD48; TNFSF10; F2RL2; CA2; SWAP70*	Glioma
Interferon alpha response	97	1.69e–04	8.45e–04	*MX1; IFI44; IFIH1; BST2; LY6E; C1S; GMPR; CASP8*	Glioma
Hallmark angiogenesis	36	1.34e–03	2.24e–02	*SERPINA5; VTN; APOH; CXCL6*	RCC
Hallmark estrogen response late	200	3.93e–04	9.82e–03	*SCNN1A; PKP3; SERPINA5; SFN; TOB1; BATF; MEST; LAMC2; CD9; PTGER3*	RCC

The top enriched terms from the well-predicted genes (“GoodPredSet”) in glioma and RCC samples are shown. The gene set enrichment analysis was performed using the Hallmark gene sets from the Molecular Signatures Database.

For KIRC, we also divided the genes into two groups, one group consisting of genes from cluster 2, 3, and 4 (GoodPredSet), and the other group from the remaining two clusters (BadPredSet). The GoodPredSet genes are enriched in the angiogenesis process while the BadPredSet genes are not (Table 1). Angiogenesis, an important factor for cancer development and progression, can change the morphometry of cells. Both morphological aspects such as vascular density and patterns as well as biological aspects such as expression of angiogenic factors have been correlated with cancer outcome.^35^

### Feature importance analysis

**Fig. 6:**
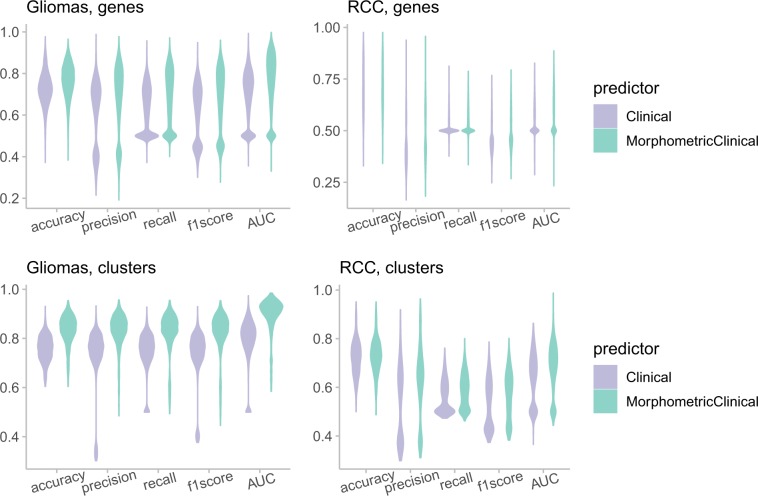
Morphometric features greatly improves gene methylation prediction compared to clinical features. The violin plot shows the comparison of model performance using clinical features only, versus using both morphometric and clinical features.

Since DNA methylation is associated with clinical features especially age, we compared the performance of the models using only clinical features (age, sex, and stage), versus using both clinical and morphometric features (Fig. 6). Adding morphometric features greatly improves the model performance compared to clinical features alone, for both cancer types.

**Fig. 7:**
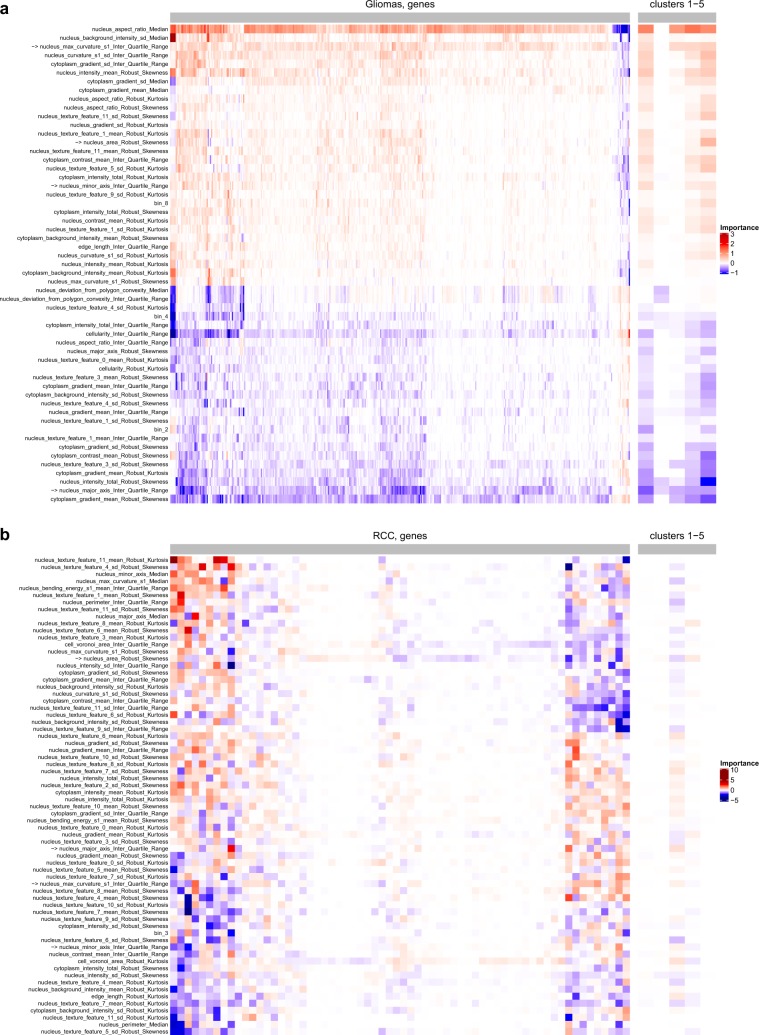
Feature importance analysis in the glioma and RCC samples. The top 25% most influential features in the logistical regression models are shown for the glioma samples (**a**) and RCC samples (**b**). For each cancer datasets, the well-predicted genes (the left side of the heat map) and the gene clusters (the right side of the heat map) are included in the analysis. Four of the most important features that are found in both the glioma and RCC samples are labeled with an asterisk.

The importance of each feature in the logistic regression model was ranked and the top 25% most important features were plotted in Fig. 7. The most important and recurrent features, including “nucleus max curvature s1 interquartile range”, “nucleus major axis interquartile range”, “nucleus minor axis interquartile range”, and “nucleus area robust skewness”, were found in both the glioma and RCC cohorts, which suggests that these features are relevant to the task of predicting the DNA methylation states.

## Discussion

In this study we demonstrated that DNA methylation states of genes in cancer can be predicted by morphometric features from whole slide images of tumor samples. We applied MethylMix, a tool to identify methylation driver genes in cancer and also infer the methylation state of a gene: hyper, hypo, or normal methylation. We have previously shown that this discrete representation of the methylation data has better performance and essentially denoises the raw methylation data.^8^ The output of MethylMix is not restricted to genes, but includes single CpGs or clusters of CpG sites, depending on the hierarchical clustering of single CpG sites within and near the genes. We tested several classical machine learning models in two example cancer data sets, glioma and RCC. In glioma samples, the models achieved an average AUC and *F*1 score of 0.74 and 0.64, respectively, for the methylation driver genes identified by MethylMix analysis. The performance can be further improved if the genes are clustered into distinct methylation groups. In RCC samples, the performance of the models was not as good as in glioma samples and fewer genes can be predicted by morphometric features. This could be partly explained by the different heterogeneous levels of the tumor samples included in the two different cohorts. Nevertheless, several genes can still be well-predicted, including the gene, *LCP1*, which is a biomarker for identifying early stage RCC.^31^ The different performance of DNA methylation prediction task can also reflect the nature of different cancers, that in glioma samples, the DNA methylation state affects the cell morphology to a greater extent.

Epigenomic mechanisms such as DNA methylation and histone modifications are crucial for gene expression and regulation. They are involved in numerous cellular processes such as differentiation, development, and tumorigenesis.^1,2^ High-throughput DNA methylation assays have been used broadly in cancer research, generating vast amounts of genome-wide DNA methylation measurements. However, array-based and sequencing-based genome-wide DNA methylation profiling assays can be expensive, require long turnaround time, and often can only provide an average measurement of the tissue samples. Thus, our study provides a rationale of associating DNA methylation states of key genes in cancer samples with the more accessible whole-slide images in clinical settings.

Integration of medical or tissue images with molecular data has been emphasized in biomedical research. For example, we constructed a multimodal neural network-based model to predict the survival of patients for 20 different cancer types using clinical data, mRNA expression data, microRNA expression data, and histopathology whole slide images.^36^ A recent study reported a deep learning model applied to whole slide images from lung cancer samples can classify lung tissues into adenocarcinoma, squamous cell carcinoma, and normal lung tissues. Furthermore, six of the most commonly mutated genes in lung cancers, *STK11, EGFR, FAT1, SETBP1, KRAS*, and *TP53* can be predicted from the images.^37^ Apart from the prediction of patient outcome, molecular features of cancer cells can also be reflected from digital images. Microsatellite instability (MSI) status of colon cancer can be predicted via radiomic analysis of computed tomography (CT) images, which adds specificity to clinical assessment and could contribute to personalized treatment selection.^38^ Another study applied deep residual learning to predict MSI directly from histology images of gastrointestinal cancers.^39^

Our study further extends the area of computational analysis of whole slide images to DNA methylation prediction. We show that morphometric information from whole slide images of tumor samples can be used to predict DNA methylation states of genes and gene clusters, which can provide insights into the underlying molecular basis of tumorigenesis. The well-predicted genes are enriched in key cancer pathways including hypoxia and cell cycle regulation in gliomas, and the angiogenesis process in RCC samples. Out of the well-predicted genes, *CDK4, MMP7, MYCBP*, and *TMEM59* in gliomas and *LCP1* in RCC have been implicated in multiple cancer types. The other well-predicted genes, including *COL5A1, CPA4, CSTB*, and *PPIC* in gliomas and *DAK, ITPRIP, TM4SF19, TMEM200A* in RCC have not been studied thoroughly in cancer and their roles in cancer worth further investigating.

The different results between glioma and RCC may be due to the fact that the tissue of origin of the two cancer sites is very different and that DNA methylation pattern is affected by tissue of origin to a great extent.^40^ Besides, the number of MethylMix genes identified for both cancer sites is very different. Since only 366 genes are identified by MethylMix in RCC, compared to 927 in glioma, we speculate that glioma are more epigenomically heterogeneous than RCC, further explaining why the results differ between the two cancers.

We hypothesize that DNA methylation can be reflected by whole slide images and that DNA methylation affects cellular morphology in several ways. Firstly, DNA methylation is shown to reflect the spatial organization of chromatin in different cell.^41^ Another study showed that CpG methylation significantly altered local DNA shape.^42^ DNA methylation is closely linked with the occupancy patterns of an important genome regulator, CTCF, which binds to insulator regions in genomic DNA and plays a fundamental role in controlling higher order chromatin structure and gene expression.^43^ To decipher whether CTCF binding plays a role in the link between DNA methylation and cell morphological changes, more comprehensive DNA methylation datasets including noncoding regions such as bisulfite sequencing together with image data are needed to expand our work. DNA methylation also reflects cell identity,^40^ therefore it follows that DNA methylation changes could correspond to different cell type mixes and thus show in the morphometric features from whole slide imaging. More importantly, DNA methylation changes in key driver genes in cancer will lead to deregulation of these genes that result in transcriptomic and proteomic alterations.^13^ These changes will subsequently influence important cellular processes including cell-cycle regulation, metabolism, and angiogenesis, which may cause morphological changes that are substantial enough to be reflected in whole slide images.

Our work has the following implications. First of all, we showed that DNA methylation states of cancer genes and morphometric features from whole slide images of tumor samples are associated. If in practice only one type of data is available, it is possible to make predictions about the other. Secondly, if both molecular and imaging information are available for building models for clinical decision support, it is important to take into consideration the association between the features from the two data types. Lastly, our results also reveal several key genes whose DNA methylation state are well-predicted by morphometric features in glioma and RCC. Further investigation of these genes might unravel new mechanisms in cancer development. The limitation of this study is that only limited cancer samples from two cancer sites are evaluated. Although we have utilized several strategies to avoid overfitting, whether the approach can be generalized to more cancer cases or other cancer sites remains to be investigated. Including other cancer types will further provide insights between DNA methylation and whole slide images, which will be addressed in future studies. We have chosen two cancer sites, glioma and renal cell carcinoma, that are very different in terms of their tissue of origin to show this broad applicability.

In summary, our results underline the potential of associations between tumor tissue as visualized on whole slide images with underlying DNA methylation states, providing new insights and understanding of how tumors develop at multiple scales.

## Methods

### DNA methylation analysis

We obtained the Infinium Human Methylation 450K DNA methylation data from National Cancer Institute (NCI) Genomic Data Commons (GDC). A total of 932 patients from the TCGA glioma cohort and 519 patients from the clear cell renal cell carcinoma cohort were included in the analysis.

Next, we used MethylMix to identify DNA methylation driver genes in cancer that are differentially methylated compared to normal samples and are also transcriptionally predictive.^7–9^ The output of MethylMix consists of a matrix of differential methylation (DM) values, which represent three classes: hyper-methylated, hypo-methylated, and normally-methylated, corresponding to positive, negative, and zero DM-values, respectively. The majority of the genes in the data sets present two different methylation states across the patients. We filtered out the genes in which one methylation state dominates more than 90% of the patients to avoid data imbalance. We also excluded the genes that present only one or more than two methylation states to focus solely on binary classification and deploy uniform evaluation strategies.

Next, given our data sets include multiple genes, we used clustering algorithms to group genes according to their methylation states. In particular, we applied hierarchical clustering and assessed the clustering quality using visual heat map inspection and the silhouette score metric.^44^ Once the genes have been assigned to the clusters, we calculated the clusters’ DM-values as the average DM-value of the genes within each cluster. Finally, we discretized the clusters’ DM-values via a Gaussian mixture model which determines an optimal discriminative threshold between high and low methylation states of DM-value clusters.

### Histopathology image data analysis

The whole slide images of cancer samples were obtained from the GDC data portal. We used the morphometric features extracted from the glioma and RCC samples with the computational methods described in the ref. ^16^ We keep the samples where both DNA methyaltion data and morphometric features are available. The features characterizing the cellular composition and heterogeneity of the histopathology images include standard cellular morphometric features and higher-level contextual summarization features. For each patient, 35 cellular morphometric features (Supplementary Table 1) and eight contextual features were extracted from their histopathology slides. The morphometric features were further summarized into mean, median, standard deviation, skewness, kurtosis, and interquartile range values.

### Multivariate modeling

A total of 342 patients from the glioma cohort and 326 patients from the RCC cohort, where both molecular and morphometric data are available, were included in the analysis. We applied machine learning models to predict the aforementioned DM-values using the morphometric features extracted from histopathology images. For each gene or gene cluster, a model is trained to predict the DM-value representing the methylation state using the morphometric features (Fig. 1). This process can be viewed as a multitask and multivariate classification problem where each task represents a gene or a gene cluster and each variable represents a morphometric feature. In the following sections, either the genes or the gene clusters are referred to as tasks. We generated 30 sets of training/testing data sets. For each set, we divided the data set into a training set (75%) and a testing set (25%). Several binary classifiers were then fitted on the training set and the best parameters are selected using a 5-fold cross-validation procedure. Due to the high dimensionality of the feature space and the relatively low number of samples, the models were regularized to avoid overfitting. The regularization parameters were also optimized through the 5-fold cross-validation procedure. We applied the following models using Python’s Scikit-learn package:^45^ Logistic Regression with lasso regularization,^46^ Random Forest,^47^ where the number of trees is optimized, Support Vector Machines^48^ where the kernel type (“linear” or “rbf”) and the regularization constant are optimized, Adaboost, where the learning rate and the number of estimators are optimized, Naive Bayes, and a two-layer Fully-Connected Neural Network with the learning rate as hyper-parameters.

### Model evaluation

We first evaluated every task-specific binary classifier using the following metrics: accuracy, precision, recall, *F*1-score, receiver operating characteristics curve, AUC score, and precision-recall curves. The scores across 30 training/testing data sets were summarized and averaged for each task. In the case of logistic regression, we also performed feature importance analysis to rank the influence of every morphometric features on the prediction task.

### Reporting summary

Further information on research design is available in the Nature Research Reporting Summary linked to this article.

## Supplementary information

Reporting Summary

Supplementary Information

## Data Availability

The data used in this study is available at: github.com/zhengh42/ImagingMethylationPrediction/tree/master/input.

## Code availability

The code used in this study can be found at: github.com/zhengh42/ImagingMethylationPrediction.
