# Supplementary material for: Whole slide images reflect DNA methylation patterns of human tumors
Source: NPJ Genom Med. 2020 Mar 10;5:11. doi: 10.1038/s41525-020-0120-9 (PMC7064513; doi:10.1038/s41525-020-0120-9)
Supplement: Supplementary file 2 — Supplementary Information [file 41525_2020_120_MOESM2_ESM.pdf]

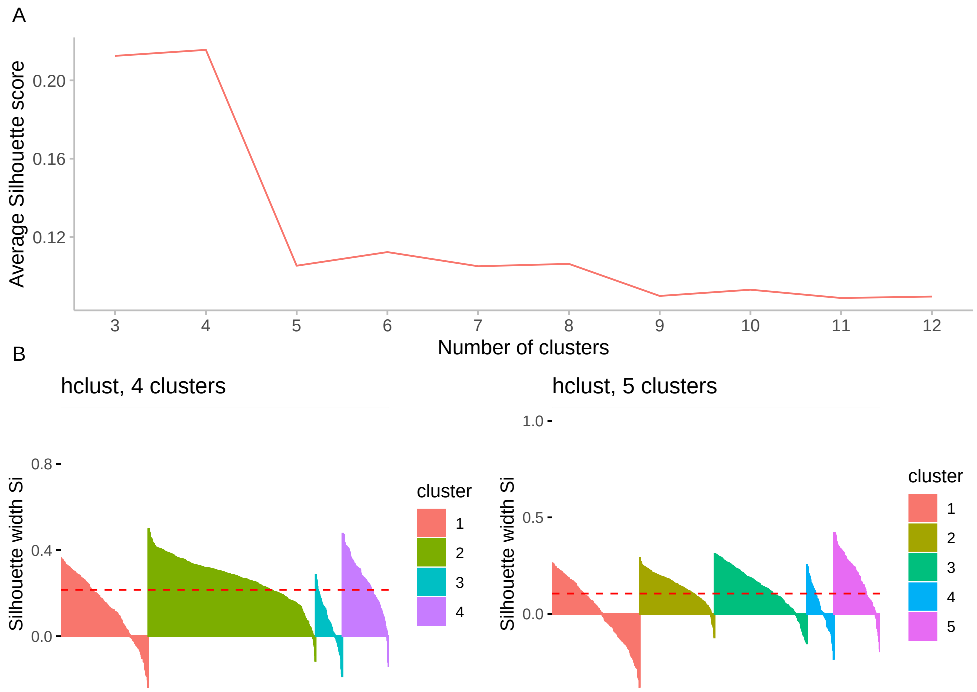


Supplementary Figure 1: Silhouette analysis for clustering in the glioma cohort.

| **List of features** |
|---|
| cell voronoi area |
| cellularity |
| cytoplasm background intensity |
| cytoplasm contrast |
| cytoplasm gradient |
| cytoplasm intensity |
| cytoplasm intensity total |
| edge length |
| nucleus area |
| nucleus aspect ratio |
| nucleus background intensity |
| nucleus bending energy s1 |
| nucleus contrast |
| nucleus curvature s1 |
| nucleus deviation from polygon convexity |
| nucleus gradient |
| nucleus intensity |
| nucleus intensity total |
| nucleus major axis |
| nucleus max curvature s1 |
| nucleus minor axis |
| nucleus orientation |
| nucleus perimeter |
| nucleus texture feature 0 |
| nucleus texture feature 1 |
| nucleus texture feature 10 |
| nucleus texture feature 11 |
| nucleus texture feature 2 |
| nucleus texture feature 3 |
| nucleus texture feature 4 |
| nucleus texture feature 5 |
| nucleus texture feature 6 |
| nucleus texture feature 7 |
| nucleus texture feature 8 |
| nucleus texture feature 9 |

Supplementary Table 1: Morphometric features extracted from histopathology images of tumors
